# Identification of exon 19 and 21 mutations of *EGFR* gene in Chinese patients with esophageal squamous cell carcinoma

**DOI:** 10.1186/1477-7819-11-266

**Published:** 2013-10-09

**Authors:** Yong Cui, Dong Chang, Mingliang Liu, Changjin Lin, Baojian Zhao, Xu Zhang, Min Gong

**Affiliations:** 1 Department of Thoracic and Cardiovascular Surgery, Beijing Friendship Hospital, Capital Medical University, Beijing 100050, China; 2 Molecular Pathology Center, The General Hospital of The Air Force P.L.A, Beijing 100087, China

**Keywords:** Epidermal growth factor receptor, Esophageal squamous cell carcinoma, Denaturing high performance liquid chromatography

## Abstract

**Background:**

Although epidermal growth factor receptor (*EGFR*) inhibitor treatment showed modest response in several clinical trials in esophageal squamous cell carcinoma (ESCC) patients, it has been reported that the frequency of *EGFR* mutations varied largely. The aim of this study was to investigate the existence of *EGFR* mutations in Chinese esophageal squamous cell carcinomas.

**Methods:**

Formalin-fixed paraffin-embedded surgically resected tumor samples were obtained from 127 randomly selected Chinese patients with ESCC. The most common *EGFR* mutations, including in-frame deletions in exon 19 and base substitutions in exon 21, were detected by denaturing high performance liquid chromatography (DHPLC) and direct sequencing simultaneously. *K-RAS* mutations in codons 12 and 13 were detected by direct sequencing.

**Results:**

In this study, L858R missense mutations of the *EGFR* gene were found in 8 out of 127 patients (6.3%) by DHPLC but no mutation was observed by direct sequencing. In addition, *K-RAS* mutation was detected in 2 out of 127 (1.6%) patients by direct sequencing.

**Conclusions:**

The incidence of *EGFR* mutations was relatively high using DHPLC method but no mutation with direct sequencing in Chinese ESCC patients.

## Background

Esophageal squamous cell carcinoma (ESCC) is the major histological type of esophageal cancer and is one of the most aggressive malignant tumors in China [1]. Despite remarkable advances in multimodal therapies, patient prognosis remains poor, even for those whose carcinomas have been completely resected [2,3]. The limited improvement in treatment outcomes by conventional therapies urged us to seek innovative strategies for treating ESCC, especially those that are molecularly targeted. One of the most promising targets is the inhibition of the epidermal growth factor receptor (EGFR) by monoclonal antibodies (for example, cetuximab, panitumumab) or small molecule tyrosine kinase inhibitors (for example, erlotinib, gefitinib) [4,5]. The EGFR is a member of the ErbB receptor tyrosine kinase family and plays an important role in cell cycle progression, angiogenesis, metastasis, and protection from apoptosis. Studies have showed that the kinase domain mutations of the *EGFR* gene in the non-small-cell lung cancer (NSCLC) tissues correlate with clinical responses to gefitinib. Most of the mutations were located in exons 19 and 21 of the *EGFR* gene including in-frame deletions in exon 19 and amino acid substitutions in exon 21 [4-6]. On the other hand, V-Ki-ras2 Kirsten rat sarcoma viral oncogene homolog (K-RAS) is a critical downstream effector of the EGFR pathway. *K-RAS* mutations are associated with intrinsic tyrosine kinase inhibitor (TKI) resistance in patients with lung cancer [7,8]. Thus, molecular diagnosis of these mutations is increasingly important in making therapeutic decisions.

Phase I and II clinical trials of the small-molecule TKIs of EGFR, erlotinib and gefitinib, in ESCC treatment are being carried out and modest activity has been observed in patients with esophageal cancers [9-11]. However, it remains unclear whether *EGFR* mutations in esophageal cancer predict benefits from treatment with EGFR inhibitors. Several studies have investigated the status of *EGFR* mutations in esophageal carcinoma and appear to suggest that *EGFR* mutations in esophageal carcinoma are rare but do exist [12-16]. Among these, one report carried out in Chinese patients found *EGFR* mutations in 14% of tumors, which is relatively higher than other regional research results. Furthermore, the authors used the scorpion amplification refractory mutation system (Scorpion-ARMS), a high sensitivity method for the identification of mutations. Therefore, it is worthwhile exploring whether different etiological factors or sensitivity methods contributed to the higher frequency of *EGFR* mutations in ESCC [16]. In this study, we investigated the existence of hot spot mutations in exon 19 and 21 of *EGFR* in Chinese ESCC patients with another sensitive method based on denaturing high performance liquid chromatography (DHPLC) as well as direct sequencing, simultaneously, and screened the status of *K-RAS* gene (codon 12/13) mutation by direct sequencing as well.

## Methods

### Patients

A total of 127 consecutive patients with ESCC who were undergoing curative resection at Beijing Friendship Hospital of Capital Medical University between April 2008 and December 2011 were enrolled in this study. Tumor staging was done by the American Joint Committee on Cancer Staging Manual (7th edition). Written informed consent was obtained from each subject, and the study procedures were approved by the institutional review board of Capital Medical University.

### DNA extraction

DNA was extracted from the tumor tissue sections (5-μm thickness) micro-dissected from formalin-fixed paraffin-embedded tumor specimens. Genomic DNA was isolated by digestion with proteinase K, followed by phenol-chloroform extractions [17].

### PCR and DNA sequencing

Three pairs of primers targeting exons 19 and 21 of the *EGFR* gene, as well as the *K-RAS* gene, were designed by using Primer Premier 5.0 (PREMIER Biosoft International, CA, USA). The sequences were as follows: exon 19: forward 5′- TGGTAACATCCACCCAGAT-3′, reverse 5′- CAGAGCAGCTGCCAGACATGAG-3′; exon 21: forward 5′- TACAGTGGATATAGAAAGGAC-3′, reverse 5′- TGCTTATTTCATCTCAATCCTACGCTT-3′; K-RAS: forward 5′- CGCCGTTAACCTTATGTGTGACATGTTCTAA-3′, reverse 5′- CGCCGCTTTATCTGTATCAAAGAATGGTCCT-3′. PCR amplification was carried out on an ABI 9700 PCR thermal cycler (Applied Biosystems, Foster City, CA, USA) in a 50 uL reaction system containing 1 × buffer (10 mM Tris–HCl (pH 8.3), 1.5 mM MgCl_2_, 50 mM KCl, and 0.1% gelatin/ml), 200 mM each of the four deoxynucleotide triphosphates, 0.5 mM of each primer, 5% DMSO (Sigma-Aldrich, St. Louis, MO, USA), 1 unit of Taq polymerase (Takara Bio, Hotsu, Japan) and 100 ng template. The PCR cycling conditions consisted of an initial denaturation step at 94°C for 5 minutes, followed by 35 cycles of 94°C for 30 seconds, 60°C for 30 seconds, 72°C for 30 seconds, and a final extension step at 72°C for 10 minutes. Reaction products were direct sequencing with an ABI PRISM 3100 sequencer (Applied Biosystems) following the manufacturer’s protocol.

### Denaturing high performance liquid chromatography-based method for the detection of *EGFR* exon 19 and 21 mutations

*EGFR* exon 19 deletion mutations were analyzed using DHPLC as described previously [18]. The most common mutation, L858R in exon 21 of *EGFR*, was detected using the restriction enzyme enriched mutation method as described except replacing polyacrylamide gel electrophoresis with DHPLC in the analyzing process [19]. Similar to Scorpion-ARMS, the detection sensitivity of the DHPLC method could reach approximately 1% mutant alleles [18,19].

## Results

### *EGFR* exons 19 and 21 mutation in esophageal squamous cell carcinoma

**Figure 1:**
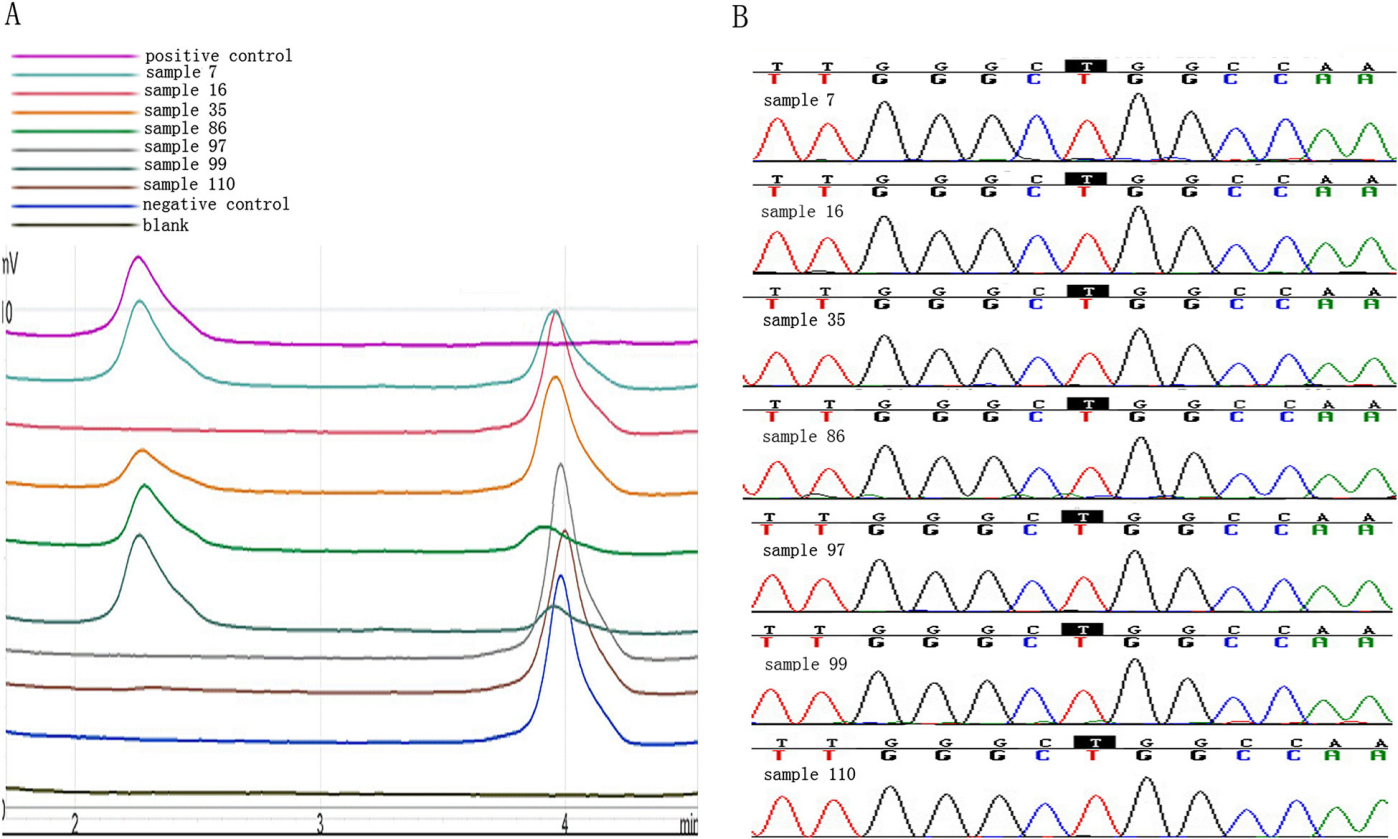
**Representative result of denaturing high performance liquid chromatography and sequencing for *EGFR *L858R mutations. (A)** L858R missense mutations in exon 21 were found using the denaturing high performance liquid chromatography-based method. A single peak on the left (123 bp) indicates mutant alleles, and that on the right indicates wild-type alleles at about 138 bp. **(B)** No mutations were detected under sequencing conditions.

No mutations in exons 19 and 21 of the *EGFR* were observed in the 127 patient tumor samples using direct sequencing analysis. However, a total of 8 samples out of 127 detected the same *EGFR* mutation (p.L858R) in exon 21 when DHPLC-based high sensitive methods were performed to detect *EGFR* mutations (Figure 1). No mutation was detected in exon 19 by either method.

### *K-RAS* mutation in esophageal squamous cell carcinoma

**Figure 2:**
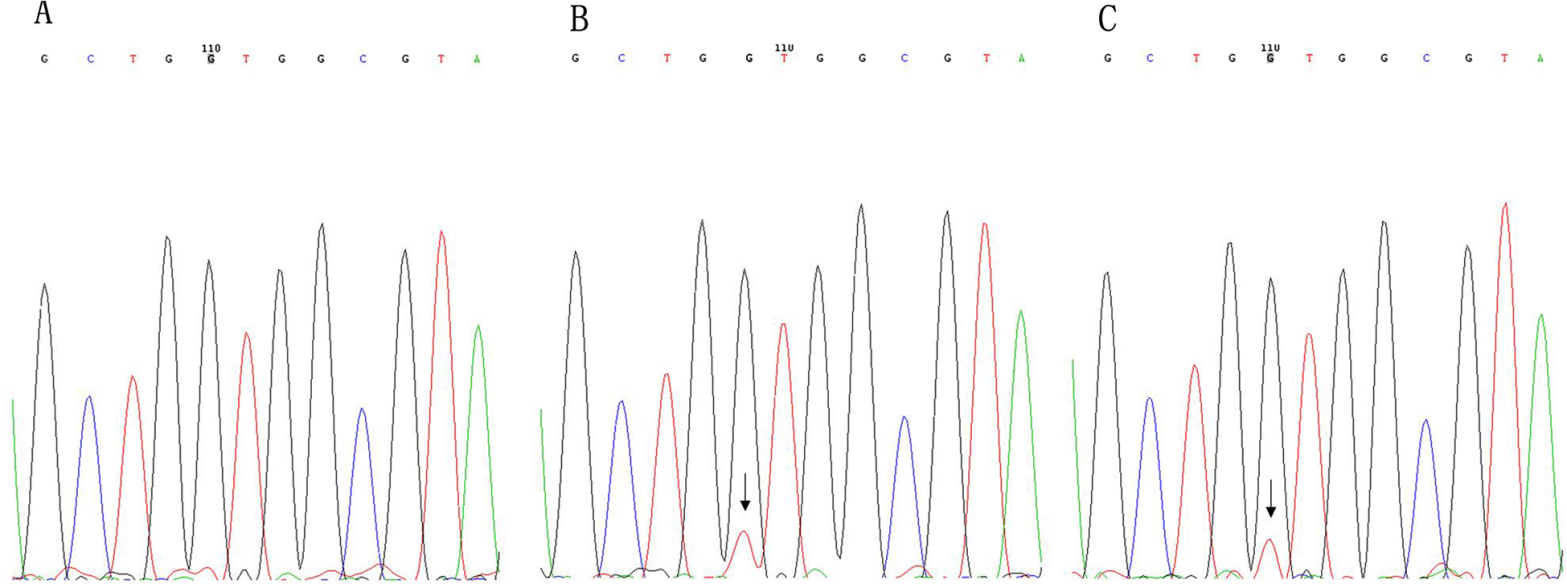
**Mutations in the *K-RAS *gene in esophageal squamous cell carcinoma samples. (A)** No mutation in codons 12 and 13. **(B, C)** Heterozygous GGT → GTT mutation in the second position of codon 12 in two samples (arrow).

A heterozygous mutation of the *K-RAS* gene (c.35 G > T; p.Gly12Cys) was detected in 2 out of the 127 patients (1.6%) by sequencing analysis (Figure 2), despite low level mutations. No mutation was found in codon 13.

## Discussion

In NSCLC, a growing number of studies demonstrated that patients with *EGFR* mutations, mainly deletions in exon 19 and L858R mutation in exon 21, would benefit from EGFR-TKI treatment, particularly among those of Asian ethnicity [5,18,20]. Furthermore, a few clinical studies of advanced esophageal cancer treatment by gefitinib showed moderate responses [9,11,21]. However, several studies have investigated the status of *EGFR* mutations in esophageal carcinoma and they mostly showed a very low frequency of EGFR-activating mutations [12-16,22-24]. It should be noted that *EGFR* mutations were detected by a high sensitive method instead of direct sequencing only in a few studies, and one of them reported relatively higher frequency of *EGFR* mutations in 14% of tumors including G719X missense mutation (n = 1), in-frame deletion (n = 2), and L858R missense mutation (n = 5) [16].

In this study, a high sensitivity DHPLC-based method, as well as conventional direct sequencing, were performed to screen deletions in exon 19 and L858R mutation in exon 21 of the *EGFR* gene in 127 Chinese ESCC patients, respectively. The results showed that 7% of the ESCC samples harbored *EGFR* mutations detected by DHPLC compared with no observed *EGFR* mutation by direct sequencing, which may be partly attributed to the high sensitivity of DHPLC in mutation detection. Our findings were consistent with a previous study in which Scorpion-ARMS, another high sensitivity method to detect *EGFR* mutation, was performed to screen *EGFR* mutation in Chinese ESCC patients [16]. Furthermore, the status of *KRAS* gene mutationwas detected by direct sequencing and showed relatively low frequency (1.6%); this was in line with previous studies in which the incidence of *K-RAS* gene mutations ranges between 0 and 16% [16,25,26]. Together with other findings, our data indicated that *EGFR* mutations exist in esophageal carcinoma at low levels, which is difficult to detect by conventional DNA sequencing. This partly explains the variant frequency of *EGFR* mutations in several studies with different sensitivity methods and complicates the efficacy of targeted therapies in some patients except for etiological factors [13,16,22]. The existence of low levels of *EGFR* mutation in ESCC indicates the presence of intra-tumor *EGFR* mutational heterogeneity, suggesting high sensitivity method should be preferred for studies exploring the correlation between *EGFR* mutations and TKI treatment in ESCC patients.

## Conclusion

Our findings demonstrated that the incidence of *EGFR* mutations in Chinese patients with ESCC was relatively higher than that of previous reports, partly as a result of mutation detection with a high sensitivity method. In line with other studies, it seemed that a high sensitivity method should be preferred when the status of *EGFR* mutations need to be explored in clinical trials of TKI in ESCC treatment.

## Abbreviations

DHPLC: Denaturing high performance liquid chromatography; EGFR: Epidermal growth factor receptor; ESCC: Esophageal squamous cell carcinoma; NSCLC: Non-small-cell lung cancer; PCR: Polymerase chain reaction; Scorpion-ARMS: Scorpion amplification refractory mutation system; TKI: Tyrosine kinase inhibitor.

## Competing interests

The authors declare that they have no competing interests.

## Authors’ contributions

MG drafted and finalised writing of the manuscript. YC and DC carried out the molecular genetic studies. BZ and XZ performed DHPLC analysis, ML and CL analysed the data. All authors read and approved the final manuscript.
